# Supplementary material for: Combining systems and synthetic biology for in vivo enzymology
Source: EMBO J. 2024 Sep 25;43(21):5169–85. doi: 10.1038/s44318-024-00251-w (PMC11535393; doi:10.1038/s44318-024-00251-w)
Supplement: Supplementary file 10 — Data Set EV1 [file 44318_2024_251_MOESM10_ESM.zip › EMBOJ-2024-117139R1-Data_Set_EV1-ds/Dataset_EV1_Legend.rtf]

Dataset EV1:Plasmid sequences and annotations for the plasmids and primers listed in Tables EV6-7.
