# Supplementary material for: Combining systems and synthetic biology for in vivo enzymology
Source: EMBO J. 2024 Sep 25;43(21):5169–85. doi: 10.1038/s44318-024-00251-w (PMC11535393; doi:10.1038/s44318-024-00251-w)
Supplement: Supplementary file 15 — Expanded View Figures [file 44318_2024_251_MOESM15_ESM.pdf]

Expanded View Figures

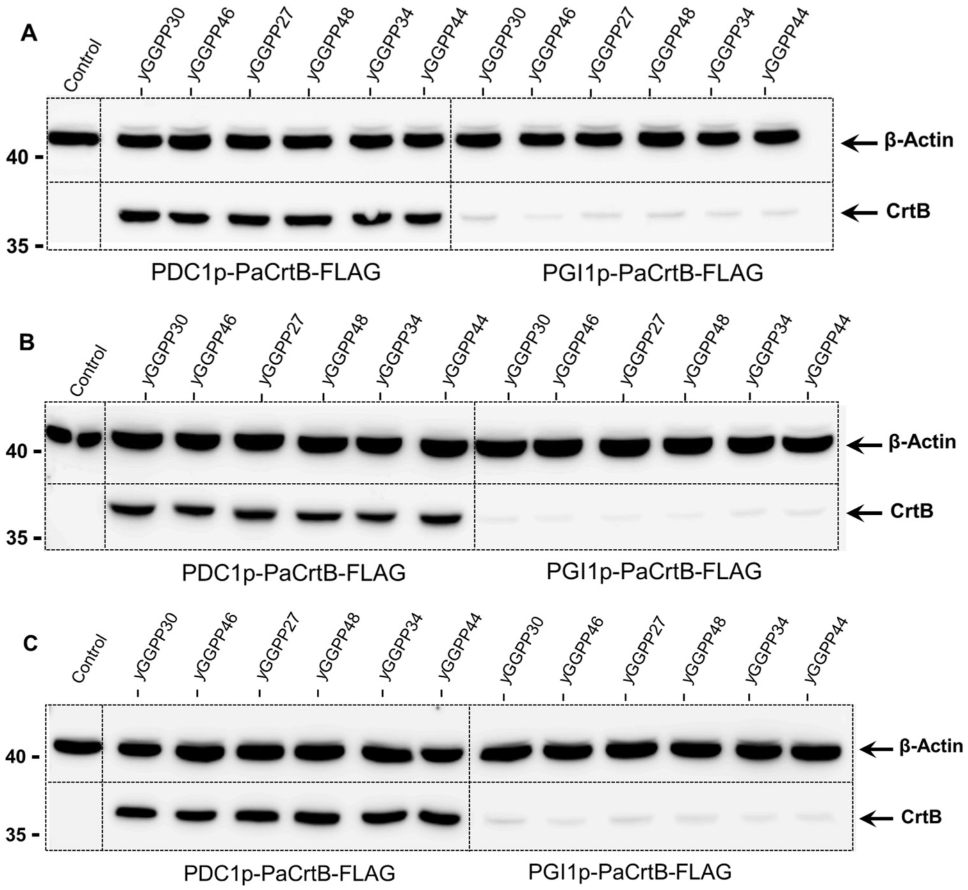

Figure EV1. Expression of PaCrtB-FLAG in the different strains.

PaCrtB-FLAG expression determined by western blot in yeast strains with different GGPP concentrations (panels (**A–C**) correspond to three different biological replicates).

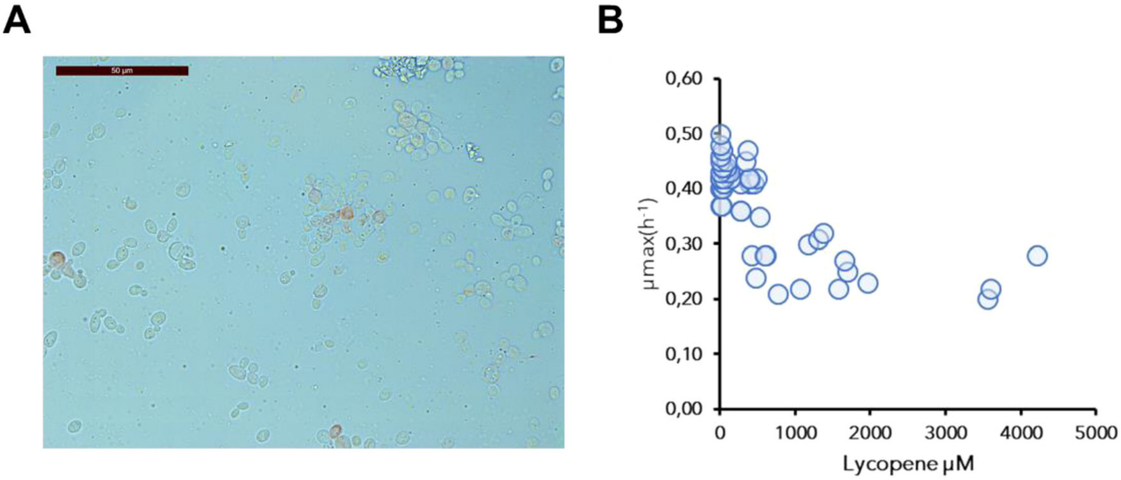

Figure EV2. Lycopene production in *S. cerevisiae.*

(**A**) *S. cerevisiae* yGGPP34 strain expressing PDC1p-PaCrtB and TDH3p-BtCrtI. Bright field images were acquired using the camera LEICA DFC300FX mounted in the microscope Leica DM4000B with Leica EL6000 light source. Lycopene crystals are observed in red. (**B**) Decrease of specific growth rate in yeast strains expressing BtCrtI with different phytoene concentrations.

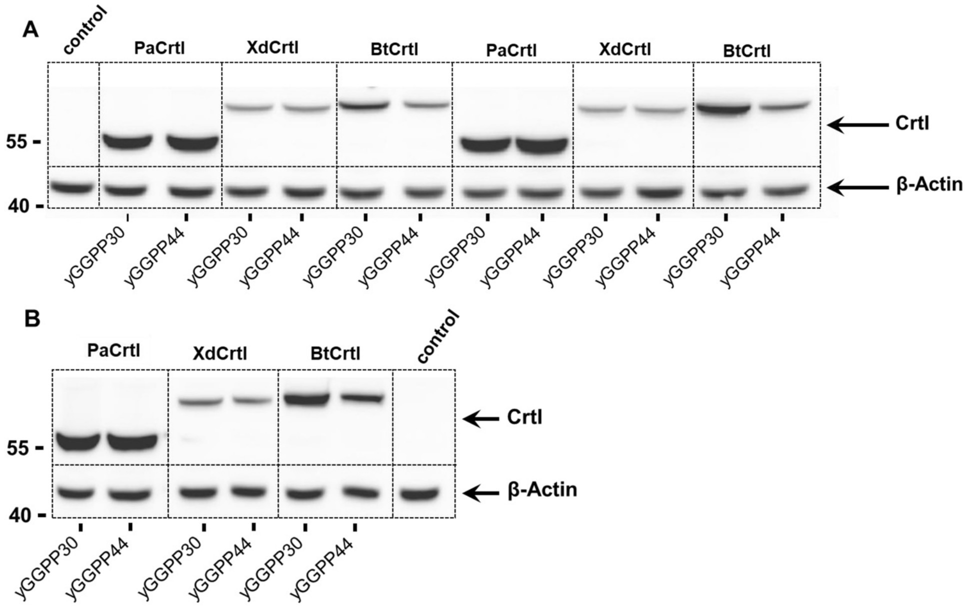

Figure EV3. Expression of CrtI in the different strains.

Expression of the three studied CrtI-V5 proteins in strains with low (yGGPP030) and high (yGGPP044) phytoene content (panels (**A** and **B**) correspond to three different biological replicates).

 © The Author(s)

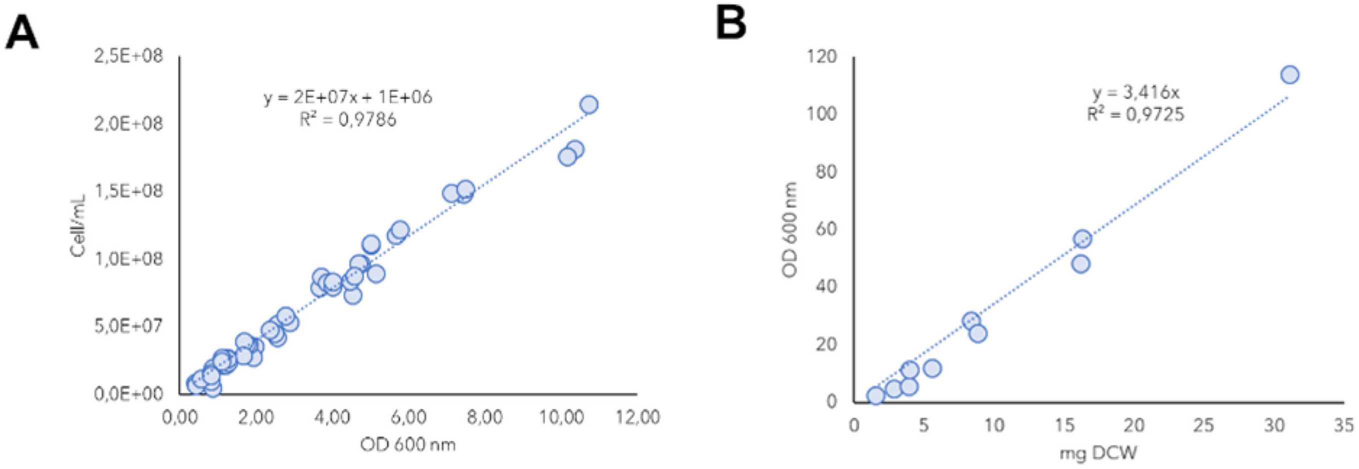

Figure EV4. Correlations between OD and biomass concentration.

Correlation between cell/mL and OD_{600nm} (**A**) and between OD_{600nm} and mg DCW (**B**).
